# *catena*-Poly[[[2-(pyridin-2-yldisulfan­yl)pyridine-κ^2^ *N*,*S*]copper(I)]-μ_1,5_-dicyanamido]

**DOI:** 10.1107/S1600536811002728

**Published:** 2011-01-29

**Authors:** Shixi Wu, Wei Jiang, Fengsheng Li, Li Liu

**Affiliations:** a National Special Superfine Powder Engineering Research Center, Nanjing University of Science and Technology, Nanjing 210094, People’s Republic of China

## Abstract

In the title compound, [Cu(C_2_N_3_)(C_10_H_8_N_2_S_2_)]_*n*_, the Cu^I^ atoms are connected by bridging dicyanamide ligands, forming chains parallel to [100]. Each Cu^I^ atom displays a tetra­hedral coordination environment, formed by one S atom and three N atoms from one 2-(pyridin-2-yldisulfan­yl)pyridine and two dicyanamide ligands. The crystal structure is stabilized by C—H⋯N hydrogen bonds, forming a three-dimensional network.

## Related literature

For potential applications of metal-organic frameworks, see: Eddaoudi *et al.* (2001 ▶). For metal-organic frameworks constructed from flexible ligands, see: Xu *et al.* (2009 ▶). For related structures, see: Mal *et al.* (2006 ▶); Schlueter *et al.* (2007 ▶); Sen *et al.* (2007 ▶).
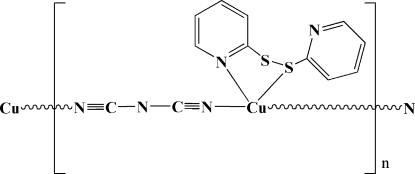


## Experimental

### 

#### Crystal data

[Cu(C_2_N_3_)(C_10_H_8_N_2_S_2_)]
*M* *_r_* = 349.92
Triclinic, 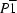

*a* = 7.6294 (15) Å
*b* = 9.5964 (19) Å
*c* = 10.202 (2) Å
α = 84.19 (3)°
β = 80.63 (3)°
γ = 70.93 (3)°
*V* = 695.6 (2) Å^3^
*Z* = 2
Mo *K*α radiation
μ = 1.87 mm^−1^
*T* = 293 K
0.20 × 0.16 × 0.12 mm

#### Data collection

Bruker APEXII CCD diffractometer
Absorption correction: multi-scan (*SADABS*; Sheldrick, 1996 ▶) *T*_min_ = 0.893, *T*_max_ = 1.000
6615 measured reflections
2669 independent reflections
2301 reflections with *I* > 2σ(*I*)
*R*_int_ = 0.018

#### Refinement

*R*[*F* ^2^ > 2σ(*F* ^2^)] = 0.028
*wR*(*F* ^2^) = 0.081
*S* = 1.07
2669 reflections
181 parameters
H-atom parameters constrained
Δρ_max_ = 0.39 e Å^−3^
Δρ_min_ = −0.29 e Å^−3^

### 

Data collection: *APEX2* (Bruker, 2007 ▶); cell refinement: *SAINT* (Bruker, 2007 ▶); data reduction: *SAINT*; program(s) used to solve structure: *SHELXTL* (Sheldrick, 2008 ▶); program(s) used to refine structure: *SHELXTL*; molecular graphics: *SHELXTL*; software used to prepare material for publication: *SHELXTL*.

## Supplementary Material

Crystal structure: contains datablocks I, global. DOI: 10.1107/S1600536811002728/zq2085sup1.cif

Structure factors: contains datablocks I. DOI: 10.1107/S1600536811002728/zq2085Isup2.hkl

Additional supplementary materials:  crystallographic information; 3D view; checkCIF report

## Figures and Tables

**Table 1:** Hydrogen-bond geometry (Å, °)

*D*—H⋯*A*	*D*—H	H⋯*A*	*D*⋯*A*	*D*—H⋯*A*
C9—H9⋯N3^i^	0.93	2.53	3.453 (3)	171

Symmetry code: (i) 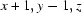.

**Fig. 1.**
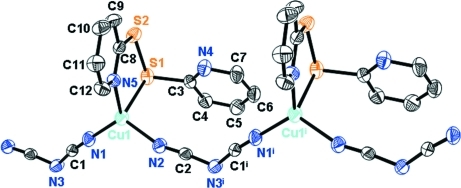
The molecular structure of a portion of the title compound, with atom labels and 30% probability displacement ellipsoids. All H atoms have been omitted [symmetry code: (i) x+1, y, z].

## supplementary crystallographic information

### Comment

Metal-organic compounds have attracted much attention because of their diverse structures (Eddaoudi *et al.*, 2001). Flexible ligands can play different roles in constructing metal-organic frameworks (Xu *et al.*, 2009). The title compound, {C_12_H_8_CuN_5_S_2_}_n,_ is constructed by two kinds of flexible ligands: briding dicyanamide ligands and chelate 2-(pyridin-2-yldisulfanyl)pyridine ligands. In this paper, the crystal structure of the title compound is presented. As illustrated in Fig. 1, the Cu atoms are connected by briding dicyanamide ligands, forming a serrate chain. Each Cu atom displays a tetrahedral coordination environment, formed by one S atom and three N atoms from one 2-(pyridin-2-yldisulfanyl)pyridine and two dicyanamide ligands, where the Cu—S and average Cu—N bonds are 2.472 (1) and 1.969 Å, respectively. The crystal structure is stabilized by C—H···N hydrogen bonds [H9···N3^iii^ = 2.53 Å, C9···N3^iii^ = 3.453 (3) Å, and C9—H9···N3^iii^ = 171°] between the central N atom of the dicyanamide and one H atom of a pyridine ring, forming a three-dimensional network [symmetry code: (iii) *x* + 1, *y* - 1, *z*].

### Experimental

CuN(CN)_2_ (0.4 mmol) and NaN(CN)_2_ (1.2 mmol) were added into 2 ml DMF with thorough stir for 6 minutes. After filtration, the colorless filtrate was carefully laid on the surface with the solution of bis(2-pyridyl)disulfide (0.5 mmol) in 5 ml *i*-PrOH. Colorless crystals were obtained after 3 weeks.

### Refinement

The H atoms were positioned geometrically and refined with a riding model, with C—H = 0.93 Å and *U*_iso_ = 1.2*U*_eq_(C).

### Crystal data

**Table:**

[Cu(C_2_N_3_)(C_10_H_8_N_2_S_2_)]	*Z* = 2
*M**_r_* = 349.92	*F*(000) = 352
Triclinic, *P*1	*D*_x_ = 1.671 Mg m^−^^3^
Hall symbol: -P 1	Mo *K*α radiation, λ = 0.71073 Å
*a* = 7.6294 (15) Å	Cell parameters from 3088 reflections
*b* = 9.5964 (19) Å	θ = 3.0–28.9°
*c* = 10.202 (2) Å	µ = 1.87 mm^−^^1^
α = 84.19 (3)°	*T* = 293 K
β = 80.63 (3)°	Block, colourless
γ = 70.93 (3)°	0.20 × 0.16 × 0.12 mm
*V* = 695.6 (2) Å^3^	

### Data collection

**Table:**

Bruker APEXII CCD diffractometer	2669 independent reflections
Radiation source: fine-focus sealed tube	2301 reflections with *I* > 2σ(*I*)
graphite	*R*_int_ = 0.018
ω scans	θ_max_ = 26.0°, θ_min_ = 3.1°
Absorption correction: multi-scan (*SADABS*; Sheldrick, 1996)	*h* = −8→9
*T*_min_ = 0.893, *T*_max_ = 1.000	*k* = −11→11
6615 measured reflections	*l* = −12→12

### Refinement

**Table:**

Refinement on *F*^2^	Primary atom site location: structure-invariant direct methods
Least-squares matrix: full	Secondary atom site location: difference Fourier map
*R*[*F*^2^ > 2σ(*F*^2^)] = 0.028	Hydrogen site location: inferred from neighbouring sites
*wR*(*F*^2^) = 0.081	H-atom parameters constrained
*S* = 1.07	*w* = 1/[σ^2^(*F*_o_^2^) + (0.0478*P*)^2^ + 0.0204*P*] where *P* = (*F*_o_^2^ + 2*F*_c_^2^)/3
2669 reflections	(Δ/σ)_max_ < 0.001
181 parameters	Δρ_max_ = 0.39 e Å^−^^3^
0 restraints	Δρ_min_ = −0.29 e Å^−^^3^

### Special details

**Table:**

Geometry. All e.s.d.'s (except the e.s.d. in the dihedral angle between two l.s. planes) are estimated using the full covariance matrix. The cell e.s.d.'s are taken into account individually in the estimation of e.s.d.'s in distances, angles and torsion angles; correlations between e.s.d.'s in cell parameters are only used when they are defined by crystal symmetry. An approximate (isotropic) treatment of cell e.s.d.'s is used for estimating e.s.d.'s involving l.s. planes.
Refinement. Refinement of *F*^2^ against ALL reflections. The weighted *R*-factor *wR* and goodness of fit *S* are based on *F*^2^, conventional *R*-factors *R* are based on *F*, with *F* set to zero for negative *F*^2^. The threshold expression of *F*^2^ > σ(*F*^2^) is used only for calculating *R*-factors(gt) *etc*. and is not relevant to the choice of reflections for refinement. *R*-factors based on *F*^2^ are statistically about twice as large as those based on *F*, and *R*- factors based on ALL data will be even larger.

### Fractional atomic coordinates and isotropic or equivalent isotropic displacement parameters (Å^2^)

**Table:**

	*x*	*y*	*z*	*U*_iso_*/*U*_eq_	
Cu1	−0.03943 (3)	0.83530 (3)	0.16964 (3)	0.05693 (13)	
S1	−0.00326 (7)	0.67390 (7)	0.37486 (5)	0.05609 (17)	
S2	0.11161 (9)	0.47767 (7)	0.28919 (5)	0.06255 (18)	
N1	−0.3026 (2)	0.9293 (2)	0.16879 (19)	0.0580 (5)	
N2	0.1131 (2)	0.9633 (2)	0.17961 (19)	0.0599 (5)	
N3	−0.6377 (2)	1.0592 (2)	0.2077 (2)	0.0694 (6)	
N4	0.3589 (3)	0.6469 (2)	0.38308 (18)	0.0610 (5)	
N5	0.1249 (2)	0.65814 (18)	0.06703 (15)	0.0464 (4)	
C1	−0.4605 (3)	0.9840 (2)	0.1839 (2)	0.0489 (5)	
C2	0.2361 (3)	1.0016 (2)	0.1905 (2)	0.0480 (5)	
C3	0.1841 (3)	0.7106 (2)	0.43562 (18)	0.0452 (4)	
C4	0.1311 (3)	0.8103 (3)	0.5330 (2)	0.0612 (6)	
H4	0.0059	0.8499	0.5684	0.073*	
C5	0.2682 (4)	0.8504 (3)	0.5770 (2)	0.0709 (7)	
H5	0.2372	0.9197	0.6414	0.085*	
C6	0.4516 (4)	0.7863 (3)	0.5242 (2)	0.0692 (7)	
H6	0.5475	0.8109	0.5519	0.083*	
C7	0.4889 (3)	0.6858 (3)	0.4302 (2)	0.0695 (7)	
H7	0.6136	0.6409	0.3963	0.083*	
C8	0.1811 (3)	0.5212 (2)	0.11824 (18)	0.0462 (5)	
C9	0.2919 (4)	0.4027 (2)	0.0454 (2)	0.0627 (6)	
H9	0.3245	0.3078	0.0850	0.075*	
C10	0.3528 (4)	0.4264 (3)	−0.0852 (2)	0.0681 (7)	
H10	0.4289	0.3484	−0.1362	0.082*	
C11	0.2996 (4)	0.5682 (3)	−0.1403 (2)	0.0660 (6)	
H11	0.3402	0.5877	−0.2292	0.079*	
C12	0.1868 (3)	0.6795 (2)	−0.0633 (2)	0.0568 (5)	
H12	0.1505	0.7748	−0.1019	0.068*	

### Atomic displacement parameters (Å^2^)

**Table:**

	*U*^11^	*U*^22^	*U*^33^	*U*^12^	*U*^13^	*U*^23^
Cu1	0.03597 (17)	0.0595 (2)	0.0745 (2)	−0.01273 (13)	−0.00278 (13)	−0.01572 (14)
S1	0.0434 (3)	0.0798 (4)	0.0469 (3)	−0.0244 (3)	0.0007 (2)	−0.0063 (3)
S2	0.0847 (4)	0.0645 (4)	0.0502 (3)	−0.0423 (3)	−0.0082 (3)	0.0048 (3)
N1	0.0393 (10)	0.0623 (11)	0.0748 (12)	−0.0157 (9)	−0.0099 (8)	−0.0125 (9)
N2	0.0394 (10)	0.0572 (11)	0.0836 (13)	−0.0155 (9)	−0.0067 (9)	−0.0086 (10)
N3	0.0382 (10)	0.0433 (10)	0.1278 (17)	−0.0077 (8)	−0.0184 (10)	−0.0154 (11)
N4	0.0464 (10)	0.0763 (13)	0.0606 (10)	−0.0187 (9)	0.0016 (8)	−0.0213 (10)
N5	0.0448 (9)	0.0463 (9)	0.0459 (9)	−0.0120 (7)	−0.0051 (7)	−0.0025 (7)
C1	0.0433 (12)	0.0430 (10)	0.0654 (12)	−0.0170 (9)	−0.0154 (10)	−0.0011 (9)
C2	0.0353 (10)	0.0381 (10)	0.0647 (12)	−0.0040 (8)	−0.0064 (9)	−0.0028 (9)
C3	0.0450 (11)	0.0532 (11)	0.0357 (9)	−0.0147 (9)	−0.0049 (8)	0.0008 (9)
C4	0.0542 (13)	0.0674 (14)	0.0528 (11)	−0.0058 (11)	−0.0028 (10)	−0.0145 (11)
C5	0.0843 (18)	0.0700 (15)	0.0601 (13)	−0.0197 (14)	−0.0148 (12)	−0.0186 (12)
C6	0.0719 (16)	0.0802 (17)	0.0667 (14)	−0.0325 (14)	−0.0237 (13)	−0.0036 (13)
C7	0.0453 (12)	0.0879 (18)	0.0762 (15)	−0.0188 (12)	−0.0066 (11)	−0.0184 (14)
C8	0.0496 (11)	0.0484 (11)	0.0467 (10)	−0.0227 (9)	−0.0093 (9)	−0.0022 (9)
C9	0.0802 (17)	0.0417 (11)	0.0665 (14)	−0.0190 (11)	−0.0118 (12)	−0.0030 (11)
C10	0.0834 (18)	0.0525 (13)	0.0635 (14)	−0.0162 (12)	0.0008 (12)	−0.0167 (11)
C11	0.0817 (17)	0.0652 (15)	0.0466 (11)	−0.0200 (13)	0.0006 (11)	−0.0081 (11)
C12	0.0596 (13)	0.0518 (12)	0.0520 (11)	−0.0108 (10)	−0.0056 (10)	0.0024 (10)

### Geometric parameters (Å, °)

**Table:**

Cu1—N1	1.9143 (18)	C3—C4	1.369 (3)
Cu1—N2	1.967 (2)	C4—C5	1.377 (4)
Cu1—N5	2.0244 (18)	C4—H4	0.9300
Cu1—S1	2.4720 (10)	C5—C6	1.373 (4)
S1—C3	1.792 (2)	C5—H5	0.9300
S1—S2	2.0207 (11)	C6—C7	1.361 (4)
S2—C8	1.787 (2)	C6—H6	0.9300
N1—C1	1.138 (3)	C7—H7	0.9300
N2—C2	1.138 (3)	C8—C9	1.378 (3)
N3—C2^i^	1.298 (3)	C9—C10	1.360 (3)
N3—C1	1.303 (3)	C9—H9	0.9300
N4—C3	1.319 (3)	C10—C11	1.375 (3)
N4—C7	1.337 (3)	C10—H10	0.9300
N5—C8	1.322 (3)	C11—C12	1.359 (3)
N5—C12	1.356 (3)	C11—H11	0.9300
C2—N3^ii^	1.298 (3)	C12—H12	0.9300
			
N1—Cu1—N2	117.16 (8)	C6—C5—C4	118.9 (2)
N1—Cu1—N5	126.58 (8)	C6—C5—H5	120.6
N2—Cu1—N5	107.60 (7)	C4—C5—H5	120.6
N1—Cu1—S1	106.75 (7)	C7—C6—C5	118.1 (2)
N2—Cu1—S1	104.29 (6)	C7—C6—H6	121.0
N5—Cu1—S1	87.90 (5)	C5—C6—H6	121.0
C3—S1—S2	105.95 (8)	N4—C7—C6	124.6 (2)
C3—S1—Cu1	102.35 (7)	N4—C7—H7	117.7
S2—S1—Cu1	98.20 (4)	C6—C7—H7	117.7
C8—S2—S1	105.52 (8)	N5—C8—C9	123.48 (18)
C1—N1—Cu1	172.06 (19)	N5—C8—S2	121.22 (16)
C2—N2—Cu1	161.63 (17)	C9—C8—S2	115.29 (16)
C2^i^—N3—C1	120.23 (19)	C10—C9—C8	119.1 (2)
C3—N4—C7	115.9 (2)	C10—C9—H9	120.5
C8—N5—C12	116.52 (18)	C8—C9—H9	120.5
C8—N5—Cu1	124.88 (13)	C9—C10—C11	118.6 (2)
C12—N5—Cu1	118.59 (14)	C9—C10—H10	120.7
N1—C1—N3	173.2 (2)	C11—C10—H10	120.7
N2—C2—N3^ii^	173.5 (2)	C12—C11—C10	119.2 (2)
N4—C3—C4	124.4 (2)	C12—C11—H11	120.4
N4—C3—S1	119.92 (16)	C10—C11—H11	120.4
C4—C3—S1	115.61 (16)	N5—C12—C11	123.1 (2)
C3—C4—C5	118.1 (2)	N5—C12—H12	118.5
C3—C4—H4	120.9	C11—C12—H12	118.5
C5—C4—H4	120.9		

Symmetry codes: (i) *x*−1, *y*, *z*; (ii) *x*+1, *y*, *z*.

### Hydrogen-bond geometry (Å, °)

**Table:**

*D*—H···*A*	*D*—H	H···*A*	*D*···*A*	*D*—H···*A*
C9—H9···N3^iii^	0.93	2.53	3.453 (3)	171

Symmetry codes: (iii) *x*+1, *y*−1, *z*.
